# Engaging and supporting Community Researchers in Low and Middle-Income Countries:  An Integrative Review

**DOI:** 10.12688/wellcomeopenres.24827.1

**Published:** 2025-10-01

**Authors:** Gill Thomson, Marena Ceballos Rasgado, Catherine Harris, Doris Schroeder

**Affiliations:** 1 School of Nursing & Midwifery, University of Lancashire, Preston, England, UK; 2 School of Health, Sport & Social Work, University of Lancashire, Preston, England, UK; 3 Health Technology Assessment Team, University of Lancashire, Preston, England, UK

**Keywords:** Community Researchers, Peer Researchers, Low-Middle-Income Countries, Systematic Review, Barriers, Facilitators, Experiences

## Abstract

The objectives of this integrative systematic review were to describe how community researchers (CRs) in low-and middle-income country (LMIC) settings are recruited, trained and supported in research projects, to identify facilitators, challenges and impacts of involving CRs, and to explore CRs’ own experiences of conducting research. Primary research studies, of any study design and in any language, that provided insights into the recruitment, training, facilitators, barriers, impacts or experiences of CRs in LMICs were included in the review. Search strategies included database searches and backward and forward chaining. Seven databases were searched on 5th November 2024 without date or language limits: Medline, Embase, CINAHL, PsycINFO, SocINDEX, Web of Science and Global Index Medicus. Quality assessment of included studies was conducted using the Mixed-Methods Appraisal Tool (MMAT). Qualitative synthesis of the findings was undertaken using a reflexive thematic approach. Overall, 39 papers reporting 27 studies were included in the review. Findings were synthesised over four themes: (1) recruitment, engagement and support; (2) benefits and challenges to the community researchers and communities; (3) benefits and challenges to the research; (4) ethics of engagement. The benefits of using CRs include facilitating access to marginalised groups, reducing power differentials between participants and research teams, and eliciting more authentic and culturally relevant data. Participation can enhance CRs’ confidence, future employment opportunities, and can foster broader positive community change. However, the findings of this review also raise concerns around ethical practices in involving CRs, the negative emotional impact on CRs, and equitable compensation, particularly in LMIC contexts where there are structural inequalities, limited resources, and sociocultural challenges. To maximise the benefits and minimise the harms, research teams must adopt more thoughtful and inclusive approaches to involving CRs in research projects, particularly around recruitment, training, support and fair remuneration.

## Lay Summary

This study presents the findings from a systematic review that aimed to identify and summarise all existing research that explores how ‘community researchers’ (CRs) – local people trained to conduct research within their own communities – are involved in research projects across low- and middle-income countries (LMICs). We wanted to find out how CRs were recruited, trained and supported and the benefits and challenges of using CRs in research. We searched for relevant studies in seven databases. Overall, 39 papers reporting 27 studies were included. We found that CRs often assist with identifying research topics, collecting and analysing data, and sharing findings with their communities. CRs can build trust, uncover issues that outsiders may overlook, and can give communities a voice in research. Many CRs gain confidence, new skills, and better job prospects. However, there are challenges when working with CRs, including limited training, emotional stress, and risks related to safety or community distrust. In LMICs, resource constraints and cultural barriers can make it harder to support these researchers effectively. Our work highlights the need for clear guidance on selecting, training, and supporting CRs to ensure their work is meaningful and ethical.

## Introduction

Community researchers (CRs) (also referred to as lay researchers or peer researchers) are typically members of a specific community, who conduct research within or about that community. This approach positions individuals with lived experiences of the topic being studied as active contributors to research. It empowers individuals within a community to participate in research on issues that affect them, challenging traditional researcher-participant hierarchies and enabling more democratic, context-sensitive knowledge production ^1^. 

CRs can be engaged at various stages of the research process, including formulating the research questions, recruiting participants, collecting and analysing data, and disseminating the findings ^1– 3^. CRs can enhance trust and cultural relevance, especially in underrepresented or marginalised communities, helping to facilitate sensitive conversations, generate grounded insights and ensure findings reflect lived realities ^1, 3^. For example, one study involved young Latin American migrant women as CRs to explore violence against women and girls within their community ^4^. Their direct involvement helped ensure that the findings reflected key concerns within their communities, such as immigration-related fears, language barriers, and cultural stigmas, issues that are often overlooked in traditional research approaches. CRs can also gain personal benefits, including skill development, and employment opportunities ^5, 6^. However, this work is not without its challenges. Tokenistic involvement and imbalanced power dynamics between academic and CRs may marginalise CRs, limit their contributions, and create harmful working relationships ^1, 7, 8^.

In low- and middle-income countries (LMICs), CRs can offer promising opportunities to address health and social inequities by enhancing cultural relevance, building trust, strengthening community capacity, and improving research engagement ^9^. However, LMIC settings may also present challenges to the effective engagement of CRs, such as resource constraints and resistance to participatory methods, particularly if they violate cultural norms ^10^. Ethical concerns may also arise when community values conflict with academic standards, particularly regarding confidentiality, or informed consent ^11^. There can also be emotional burdens associated with CRs’ sharing similar lived experiences with participants, particularly in studies involving trauma or sensitive topics ^11, 12^. While this issue is salient for all CRs, some authors argue that this issue may be more pertinent in LMICs due to working in communities with abject poverty and historical marginalisation ^13^. Maintaining confidentiality and ensuring personal safety of CRs may be particularly challenging in LMICs and CRs may experience heightened stress due to their dual identity as both insiders and researchers ^14^. Poor literacy coupled with inadequate training may also mean that CRs struggle to collect data effectively or manage emotional disclosures, which not only affects data quality but may also cause harm ^8^.

Despite growing interest in CRs, there is no comprehensive insight as to how they are involved in research in LMICs. To our knowledge, no systematic review has mapped how CRs are identified, trained and meaningfully engaged across diverse LMIC settings. Addressing this gap could help support ethical and effective participatory research in global health and development. This integrative systematic review aimed to identify and synthesise existing literature on the inclusion of CRs in LMIC research projects. Specifically, it sought to: (1) describe how CRs are selected, trained, and supported; (2) identify facilitators and challenges to their involvement; (3) examine the impacts of involving CRs - from individual to research-level outcomes; and (4) explore CRs experiences of conducting research in different LMIC contexts.

## Methods

This integrative systematic review followed the methodology outlined by Whittemore and Knafl ^15^ and is reported according to the PRISMA guidelines ^16^. A protocol was registered in PROSPERO before commencing the review (registration no. CRD42024608904).

### Search

The following databases were searched on 5 ^th^ November 2024: Medline (Ovid), Embase (Ovid), CINAHL (EBSCOhost), PsycINFO (EBSCOhost), SocINDEX (EBSCOhost), Web of Science (Science Citation Index Expanded; Social Sciences Citation Index; Arts & Humanities Citation Index; Conference Proceedings Citation Index-Science; Conference Proceedings Citation Index-Social Science & Humanities; Emerging Sources Citation Index), and Global Index Medicus (all indexes). The search strategy used keywords and synonyms related to the concept of CRs to identify relevant studies. The full search strategy used for each database can be found in Supplementary File 1 ^17^. No date, language or other limits were applied to the searches. The search results were downloaded into EndNote and duplicates were removed before importing the deduplicated records into the Rayyan software for screening. Forward and backward citation searches of included papers were conducted using Scopus to identify further relevant studies.

### Inclusion criteria

Primary research studies undertaken in any language with no time restrictions that provided insights into the recruitment, training, facilitators, barriers, impacts and experiences of CRs in LMICs were included. CRs were defined as members of the community who have received training to undertake research-related activities in their local communities. Studies that only involved community members in research, rather than actively recruiting, training and supporting them to undertake the research directly, were excluded.

Studies focusing on peer educators and citizen scientists were also excluded, as the role of peer educators ^18^ is to provide education and support to their peers on specific topics, while citizen scientists voluntarily contribute to scientific research, often on large-scale or global issues ^19^. These roles differ from community researchers, who are embedded within a specific community and focus on producing knowledge that directly addresses that community’s needs and priorities. We also excluded any studies that provided insufficient detail, e.g., only a cursory mention of including CRs and how they were trained. 

All study designs (quantitative, qualitative and mixed methods) were included but books, book chapters, commentaries, editorials and grey literature such as reports and theses were excluded. While we intended to check related systematic reviews to ensure that all relevant included studies had been identified, no relevant reviews were located.

### Study selection

Titles and abstracts of all records retrieved in the searches were independently screened by two reviewers using the Rayyan software ^20^ with disagreements resolved through discussion with a third reviewer (GT, MCR or CH). The full text papers of records meeting the inclusion criteria were then screened by a single reviewer (GT, MCR or CH) with regular meetings between all three reviewers to ensure consistency in decision making and to resolve any uncertainties.

### Data extraction

Data extraction was conducted by a single reviewer (MCR) with 20% verification by a second reviewer (CH) using a customised and pre-piloted data extraction tool in Excel. The data items extracted included: study aims, country of study, study focus, CR demographic characteristics, number of CRs involved, study design, where and how CRs were recruited, data collection methods used by CRs, involvement of the CRs in the study, and training elements.

### Quality assessment

The methodological quality of the included studies was evaluated using the Mixed-Methods Appraisal Tool (MMAT) ^21^. Quality assessment (QA) was carried out by a single reviewer (MCR) with 20% verification by a second reviewer (GT).

### Data analysis

As all the studies were qualitative, a reflexive thematic approach was undertaken ^22^. This involved line by line coding of any data that could help answer the research questions; and not coding insights into data that was outside of the review aim and objectives, such as study participants’ views about the study topic. The next step involved merging the codes into themes and associated sub-themes that represented the whole data set. We chose not to present the findings under each review question to avoid overlap - for example, when describing facilitators and challenges separately from experiences. Analysis was led by GT and then checked by MCR. The final interpretations were discussed and agreed upon by all the authors.

## Findings

**Figure 1.**
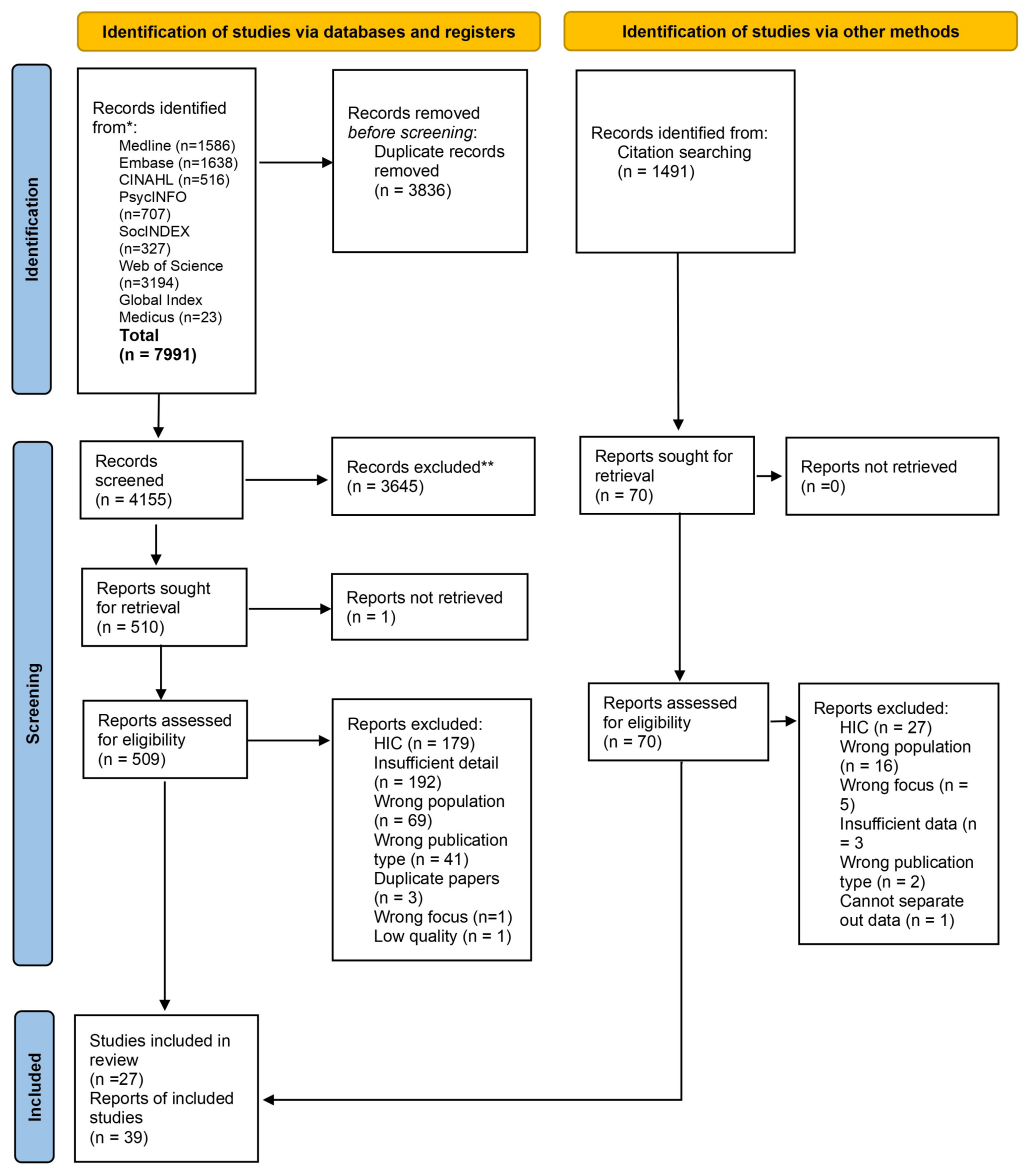
PRISMA diagram.

Overall, 7,991 records were identified from the database searches, and an additional 1,491 identified via backward and forward screening. Following removal of duplicates 5,646 were screened at title and abstract stage, 579 papers were read in full. The reasons for exclusion at full text stage included being undertaken in a high-income country (n=179) and the paper including insufficient detail (n=192). A total of 39 papers representing 27 studies were included (see Figure 1 for PRISMA diagram; see Supplementary File 2 for PRISMA checklist ^17^) in the review. Some studies ^13, 23– 39^ were reported across multiple publications, with different papers presenting various aspects of the same research. The characteristics of the studies are reported in Table 1 (see Supplementary File 3 ^17^), and details of the recruitment, involvement and training of CRs are reported in Table 2 (see Supplementary File 4 ^17^).

The quality of the included studies, assessed using the MMAT, varied considerably, with scores ranging from 0% to 100%. While many studies (n=18) achieved full scores, lower scores were generally due to a lack of methodological detail, particularly regarding the description and justification of methods and data analysis processes (see Table 1 – Supplementary File 3 ^17^).

### Summary of included studies

Most of the studies were published in the last decade (n=18) and were conducted in a range of LMICs, with the highest number of studies conducted in South Africa (n=11) (see Table 1 – Supplementary File 3 ^17^) ^12, 27, 30, 37, 40– 46^. Three studies ^10, 12, 44^ included CRs from HICs in addition to CRs from LMICs. 

In relation to the study’s aims (see Table 1 – Supplementary File 3 ^17^), ten of the included studies focussed on reporting and exploring the methods used in involving CRs in participatory research projects ^30, 31, 39, 42– 44, 47– 50^. Five studies focussed on the experiences and perceptions of CRs ^12, 37, 40, 41, 51^ and four ^36, 52– 54^ focussed on describing new methodological approaches to using CRs in research. Three studies focussed on identifying the benefits, opportunities and challenges in involving different groups as CRs ^10, 27, 45^. Four studies did not focus on how the CRs were involved in the study; however, they were considered to provide useful information on the recruitment and training of the CRs ^46, 55– 57^. One study compared the quality of data collected in focus groups led by adolescent CRs with those led by adult researchers ^58^. 

The focus of the research projects described in the studies was varied (see Table 1 – Supplementary File 3 ^17^) and included sexuality research ^10, 27, 40, 42, 48^, violence and/or mental health ^12, 46, 51, 57^, sexual health ^36, 53, 54^, mobilities of specific populations ^30– 34, 44^, environmental research ^49, 50, 55^, street-connected children and young people ^47, 56^, nutrition ^41, 43^, determinants of health ^13, 23, 24, 39^, maternal health ^52^, student’s attitudes to ethnicity and politics ^58^, HIV/AIDS, aging and child wellbeing ^45^, and a cancer prevention intervention ^37, 38^.

In the following sections, we present the synthesised findings over four themes and associated sub-themes (see Table 3). 

**Table 3.** Overview of themes and sub-themes.

Themes	Sub-themes
**Recruitment, engagement and support**	Recruitment strategies Levels of CRs’ involvement Who are the CRs? Training and supervision Reflexive and reflective approaches
**Benefits and challenges to the CRs and communities**	Social standing Altered sense of self New knowledge and understanding New opportunities Hearing the voices Fieldwork hazards Benefits to communities
**Benefits and challenges to the research**	Unheard voices Skills and capabilities of the CRs Engaging with the communities: • Relationships with community members • Research focus • Demographic-related issues and • Time and resources
**Ethics of engagement**	Ethical considerations Power differentials Paying the CRs

### Theme one: Recruitment, engagement and support

In this theme, we describe the ‘recruitment strategies’, the ‘levels of CRs’ involvement’, ‘who are the CRs?’, ‘training and supervision’ and ‘reflexive and reflective approaches’ used with the CRs.

***Recruitment strategies***

Several studies described how they recruited the CRs. The strategies included local advertisements in the communities ^39, 45^, asking representatives within local governmental and/or non-governmental (e.g., community) organisations to advertise or nominate suitable individuals ^12, 30, 38, 46– 48, 52, 53, 55, 56, 58^ or by ‘word of mouth’ ^37, 45^ (see Table 2 – Supplementary File 4 ^17^). One study that involved recruiting CRs in London and Cape Town found more challenges in London as high unemployment and networks of community organisations meant identifying CRs to be much easier in Cape Town ^44^.

Overall, recruitment procedures were rarely detailed – with some exceptions. For instance, in one study, the school-aged CRs were asked to write an essay on transport/mobility prior to their appointment as CRs ^34^. In the Keygnaert *et al.* study exploring sexual violence in sub-Saharan areas, CRs were interviewed to assess their communication skills, potential research skills, empathic attitude, social engagement and leadership capacities ^57^. Whereas in Mosavel *et al.*’s study, every person who expressed an interest was interviewed and asked questions to elicit their ‘ *mutual compatibility and transparency’* (p.4: 37). The fact that some of the CRs were later found to be unsuitable due to ‘ *incompetence*’ ^37^ would suggest that more rigorous procedures were needed.

In some studies, agreement to involve the CRs was a multi-phased process. For example, in one study this involved initially working with local statutory and non-statutory organisations to suggest individuals and then working with village leaders to formally nominate women to take part ^52^: with authors postulating how these processes were specifically designed to minimise challenges and increase the CRs’ acceptability in the community. The studies involving child or youth-based CRs generally involved parental as well as CR agreement (e.g., 34, 40, 46). Chappell *et al.* also considered that home visits to seek parental agreement provided the research team with valuable insights into the CRs’ cultural and geographical backgrounds ^40^. While the suitability of recruitment strategies was not often reflected upon, one study highlighted difficulties in obtaining buy-in from local schools to recruit CRs, and despite the University’s ‘ *excellent local connections’* (p. 158: 30).

***Levels of involvement***

The extent to which CRs were involved in research-related processes (see Table 2 – Supplementary File 4 ^17^) varied. It was generally common for CRs to develop data collection tools, such as producing, adapting or reviewing questions to be used (e.g., in surveys, interviews, focus groups, etc) ^10, 13, 30, 33, 34, 36, 37, 42, 44, 45, 47– 49, 51– 58^ – with some CRs tasked with identifying the research topics either initially or during data collection based on what was emerging ^13, 34, 45, 51, 52, 55^. In several studies, the CRs were provided with tools that could be updated and adapted (e.g., 54, 58), whereas in others, the data collection questions were co-produced with the CRs (e.g., 45, 52, 57): in Price *et al.* this meant that the final version was very different from what the study team had initially envisaged ^54^. In Elmusharaf *et al.*’s paper they provide a detailed overview of how CRs - due to literacy challenges - used drawings to develop research questions in a ‘ *wholly participatory’* process ^52^. On occasion, the decisions for which data collection tools would be used were amended based on the capacities of the CRs. For instance, in Shaw *et al.*’s project exploring renewable-energy projects in Kenya, the research team originally developed multiple approaches (e.g. capturing stories, historical timelines, resource maps and livelihood trend analysis) for the CRs to use ^50^. However, as these methods were too complex for less experienced CRs, they had to revise the ideas for a more structured approach to be used (e.g., interviews and focus groups).

Wide variations in the description and extent of CR involvement in data analysis were also noted across the studies. This could range from CRs being mentioned as being involved, but with little detail provided (e.g., 47, 49); to CRs being invited to reflect on the themes and interpretations produced by the study team (e.g., 25, 44, 45, 55, 57); to CRs developing the initial coding framework ^13, 50, 53^. In the Elmusharaf *et al.* study exploring maternal health in South Sudan, a one-day workshop was held for the CRs to discuss key issues and present their analysis of the data using role-play, followed by a discussion with the wider project team ^52^. While some authors highlighted that CR involvement in data analysis enabled richer insights to emerge ^34, 45^, other studies noted limited CR participation due to data analysis being a “ *delicate and lengthy process*” and CRs lacking the confidence or skills to fully undertake the role (e.g. 35).

Other ways in which CRs were involved in the research included contributing to sampling techniques to identify study participants ^13, 51, 53, 57, 58^, agreeing participant incentives ^57^, deciding which CRs would be involved in which data collection methods ^13, 30, 32^, data entry ^13^, deciding the location and/or timings of data collection ^12, 32, 34, 42, 51^ and translating the study materials ^13, 27, 47, 51, 56^. In some studies, CRs were reported to have contributed to developing ethical procedures ^13, 30, 44, 57^ such as ‘ *procedures for informed consent, safety and respect with participants during fieldwork’* (p.5: 44): in one study, the CRs read and provided feedback on lay summaries ^13^. 

Several studies also described CRs involvement in dissemination activities. Examples of dissemination involvement included CRs working with the research team to decide the recommendations to be shared ^13, 34, 47^: co-authoring poster presentations and journal articles ^13^; presenting the findings at community and wider stakeholder events ^10, 13, 30– 32, 34, 36, 43, 44, 47, 55^ or being interviewed for national television ^30^. In a few studies, the CRs also co-produced dissemination materials such as a book of findings ^31, 34^, comics, animations, and policy briefing papers ^47^. Some authors emphasised the importance of sharing and acting upon the findings to prevent CRs disillusionment ^30^. 

***Who are the CRs?***

Several studies provided specific details about the CRs such as detailing demographic-related information ^25, 30, 39, 57^ (see Table 1 Supplementary File 3 ^17^ for CRs' characteristics). Others just provided broad descriptions such as *‘four CRs were selected per site’* ^53^. Many studies referred to recruiting CRs based on specific eligibility criteria such as age and/or sex diversity (e.g., 30, 37, 39, 46, 52, 55), marital status ^52^, language skills ^36, 38, 52, 53^, area of residence ^30, 53^, familiarity with community members ^53^ and perceived availability ^38, 51, 52, 56^. On occasion, CRs were selected from a wider pool of potential participants ^37, 38^. For example, in Chappell *et al.*’s study this involved three out of 22 young participants being selected and trained as CRs based on specific criteria (age, disability, interpersonal skills and geographical location) ^40^. 

In some studies, CRs included individuals who were members of the traditional leadership such as community police officers ^24^. Other studies had a stronger community focus, with individuals recruited based on shared characteristics with the study participants such as having a visual or physical disability ^35, 36^, being a former street child ^56^, being a migrant ^57^, being an urban farmer ^43^, being unemployed or casually-employed young men ^44^, or being from the same social networks ^32, 42, 51^. 

In some studies, recruiting CRs based on specific characteristics meant the individuals also differed in other respects. For example, while Kana *et al.* highlighted the importance of recruiting former street children as CRs, at the time of the study these individuals were employed in different roles including a university student, taxi driver and a gardener ^56^. While a few projects referred to the CRs’ expected minimum level of schooling, suggesting a certain level of literacy was important ^31, 37, 53, 56^, this could mean having to compromise on other characteristics such as age, where finding older women who were literate was a challenge ^31^ – and potentially precluding the most disadvantaged ^54^. Some studies also had to compromise on certain characteristics due to recruitment challenges. For instance, Burke *et al.* planned to work with young people below the age of 25 years in their study exploring the barriers and enablers faced by young people with disabilities when accessing sexual and reproductive health services ^36^. As they struggled to identify sufficient young people in this age range, who also met other essential criteria (e.g.., having a disability), the age range was extended to 25–30 years.

What was rarely identified across the literature was recruitment based on attributes rather than characteristics ^40^. In one exception the CRs needed to have specific qualities such as being ‘ *committed and motivated and with a desire to have a voice and tell the story of their peers’* (p.1348: 52).

***Training and supervision***

While details on the training were largely absent from a few studies (e.g., 46, 49, 51, 58), most studies provided some detail about the length and content (see Table 2 – Supplementary File 4 ^17^). In only one study ^51^, authors explicitly described that to be eligible to conduct the research, successful completion of the researcher programme was required. 

Regarding length of training, in Brear’s study ^24^, the CRs could attend up to 96 workshops although there was no indication over what period the workshops were undertaken. Across the other studies, training was generally provided over a four or five-day period, with exceptions – one study provided training over two weeks ^37, 38^ and others over a more extended period ^10, 36^. For example, Burke *et al.* provided an initial 4-day training programme, followed by a one-day analysis workshop and then a further half day for CRs to reflect on the research processes and experiences ^36^. Some based the training on an existing framework, e.g., the EXPLORE toolkit developed by a non-government organisation ^10, 35, 36^. While a few studies discussed the research topic within the training (e.g., 10, 35, 36, 52), on most occasions the training focused on research-related processes and procedures (see Table 2 – Supplementary File 4 ^17^).

Overall, there appeared to be little consideration of the CRs' backgrounds in the development of the training programmes. Notable exceptions included instances where CRs were asked about their expectations and what they hoped to achieve through their involvement ^52^, or where training acknowledged their educational backgrounds, prior work experience, and cultural contexts ^58^. Most training programmes were conducted in-house by the research team. However, on occasion professional facilitators were involved: in one study, the external facilitator designed activities to encourage engagement, including team motivation exercises and cultural presentations ^55^.

Opportunities to practice data collection activities was a common approach across the studies, both within the training programme (such as through role plays, practice interviews, simulations with the CRs and/or wider members of the research team) and piloting within the community (see Table 2 – Supplementary File 4 ^17^). In a three-site study ^30, 32– 34^, this also involved working through different scenarios of potential field problems informed by the team’s previous experience. The Elmusharaf *et al.* study also referred to exposing CRs to good and bad interviews as a valuable learning tool ^52^. In some projects ^51^, the number of practice sessions were flexibly provided based on the skills of the CRs. 

Overall, piloting served an important purpose of ensuring the CRs had the capabilities to undertake the work, and to refine the data collection tools. For example, the CRs in Price *et al.*’s study on young people’s sexual and reproductive behaviour, suggested significant changes to the interview prompts during the training, and then for further adaptations following field-testing with community members ^54^. In Brear’s study, the CRs chose to reduce the number of sensitive and personal questions as they were unable to prevent husband’s overhearing their wives’ responses ^24^. 

Another common approach was ongoing supervision and/or debriefing opportunities. In most cases, this support was offered regularly - such as daily or weekly - throughout the data collection period (e.g., 10, 12, 13, 31, 32, 34, 37, 38, 40, 45, 48, 54, 55) to enable CRs to share their experiences and raise any challenges they encountered. In one study examining the role of armed conflicts in the motives of children to join the street in Ituri ^56^, a psychologist was present for all the interviews and after each interview a debriefing session was held with the CR; whilst the wider research team also provided individual and group supervision. In Keygnaert *et al.*’s study, the CRs participated in two coaching sessions ^57^. During these sessions, they submitted the completed interview guides and received the agreed remuneration. Some projects also offered the flexibility of daily contact (via phone or apps) (e.g., 10, 35, 36, 51, 55). On occasion, the CRs were reported to seek support from one another, or from other local stakeholders (e.g., teachers) ^32^. 

At the end of the study, CRs in several projects were awarded certificates in recognition of their involvement ^32– 34, 37, 38, 44, 49, 52, 57^.

***Reflexive and reflective practices***

Across the studies there was evidence of reflective or reflexive processes being introduced to ensure the findings were balanced and grounded in participants’ experiences and perspectives. For example, the CRs in Price *et al.*’s study, exploring young people’s sexual and reproductive behaviour in Zambia, were encouraged to avoid talking to certain individuals who may hold certain opinions ^54^. Some of the training programmes were specifically designed for CRs to reflect on their own views of the research topic (e.g. 10, 54) and to share and discuss divergent views collected from research participants to generate more nuanced insights ^45^. 

In several studies, CRs were encouraged to complete a journal, notebook or field diary (to reflect on data collection or emergent findings, with this information often used as data within the study) ^31, 33, 34, 36, 37, 40, 42, 44– 46, 48, 51, 53^. In Palfreyman *et al.*’s study, the CRs were given a structured journal template for data recording, and a research diary for private and instruction-free use ^51^. Whereas in Yilmaz *et al.*’s, study the young CRs used a diary to capture and reflect on their experiences through writing and/or drawing to encourage those less confident to participate in the research process ^46^. In Mosavel *et al.*’s study, journals were only introduced part way through the research process due to CRs being negatively affected by hearing study participants’ insights ^37^. 

Burke *et al.* used a number of activities to encourage reflexivity including writing i-Poems (poems that focus on first person statements to illustrate how they represent themselves in interviews), journals and daily debriefing sessions ^36^: as some CRs questioned the validity of the data, the team introduced a form to be completed after each interview asking the CRs to reflect on if and why they did not believe the respondent ^36^. In Schatz *et al.*’s project, discussing the use of CRs in different health-related projects in South Africa, the research lead met with the CRs monthly - when field notes were submitted - to discuss their emotional reactions, understandings and interpretations of the broader context, thereby contributing local insight and embedded knowledge ^45^. Whereas Paganini *et al.* introduced a 2-day reflection retreat for CRs to critically consider what the research achieved, and how their prejudices and presuppositions influenced the research ^43^. Although the CRs in one study reported needing more training to address their personal reactions during data collection ^51^.

### Theme two: Benefits and challenges to the CRs and communities

In this theme, we describe the benefits (‘social standing’, ‘altered sense of self’, ‘new knowledge and understanding’ and ‘new opportunities’) and challenges (‘hearing the voices’ and ‘fieldwork hazards’) for the CRs. The final sub-theme, ‘benefits to communities’, explores how CR involvement can generate positive impacts for the wider community.

***Social standing***

In a few studies, CRs’ involvement was believed to have enhanced their social standing in the community ^30^. This could relate to CRs’ perceived endorsement from governance authorities and links with ‘White’ academics ^24, 29, 39^. Likewise, in Robson *et al.* the authors noted that the training, association with teachers and external researchers, and possession of research tools all contributed to the young CRs being perceived as different from - and ‘above’- other children ^33^.

In Page *et al.*’s study, CRs felt that their visibility- through newspaper articles, podcasts, and social media produced as part of the dissemination strategy - gave greater weight to their views ^10^. Similarly, in Garnett *et al.*’s paper exploring natural resource management in different settings (Australia, Zimbabwe, Ethiopia, Kenya), some CRs went on to hold positions of authority within the community, such as becoming a chairperson or a member of a traditional jury ^49^. Community members also continued to seek them out for advice ^49^. Similarly, in Hampshire *et al.*’s study, this elevated social standing was reflected in CRs being seen as sources of knowledge, as one CR explained: ‘ *people in town come to ask me for advice’* (p. 226: 34).

***Altered sense of self***

Several studies reported that participation in the research had a positive impact on CRs’ sense of self-identity. In one study, this involved CRs valuing the opportunity to think for themselves, to be listened to, and to actively contribute in a safe, non-judgemental learning space – an approach very different to their previous education experiences - ‘ *no one says wrong, or no one comes with a stick and beats me’* (p. 1446: 23). In other studies, the positive experiences CRs had while engaging with members of the study team and participants were believed to have significantly impacted on their confidence and how they socialised with others ^23, 31, 34, 46, 55^. One CR stated:

At first, I was a very shy person, but because of skills in interviewing, I can approach people and talk to them freely. ... This project ... has taken my shyness away. Now I don’t feel unnecessarily shy as I used to be, and I think this will stay with me forever. The project has made me more confident in whatever I do (p.225: 34).

CRs repeatedly referred to how their involvement had developed their verbal and non-verbal communication skills ^23, 31, 32, 34, 37, 41, 42, 51, 55, 57^ leading to noted improvements in their relationships with their mothers ^37^ and everyday lives ^10, 41, 51^: ‘ *research taught me how to talk to people*’ (p.5: 32).

Because CRs shared similar characteristics with the study participants, this meant they were able to gain new perspectives and insights into their own lives ^46^. For instance, in Chappell *et al.*’s study on young people with disabilities, CRs reported that listening to participants’ experiences helped them develop a deeper understanding of issues such as sexuality and life with a disability, which in turn influenced their own sense of self ^40^.

CRs reported a sense of pride in what they had accomplished, which for some related to a renewed trust in their abilities ‘ *Now I can be on my own and do things on my own and do things on my own for me.*’ (p. 10: 37). For others, pride stemmed from the tangible outputs of the research-for example, in Hampshire *et al.*’s project, a book of findings was produced, with all CRs named and acknowledged within it ^34^. For some CRs this restored self-belief, and self-confidence gave them motivation ^37^ and hope for a different future such as *‘when* [they] *become a parent’* (p. 8: 51).

***New knowledge and understanding***

Across several studies, CRs reflected on how their participation provided deeper insight into the challenges faced within their communities. While this information could at times be emotionally difficult (discussed below), gaining an understanding of local issues ^36, 40– 44, 46, 49– 51, 55, 57^, learning about their own history and culture ^50^, or engaging with the topic focus (e.g., nutrition and healthy eating) ^41^ was often viewed as a positive and empowering experience. In a few studies, this knowledge challenged myths and stereotypes, e.g., about HIV status ^40, 51^. In Page *et al.*’s study exploring sexuality-focused issues in The Netherlands and Indonesia, some CRs reported greater acceptance of people with stigmatising experiences. In one study CRs shared how hearing participants’ stories had helped them to consider strategies they would use to cope with present or future trauma ^46^.

CRs were also reported to have developed new knowledge and skills related to various aspects of the research process. These included the value and purpose of research, teamwork, time management and data collection activities (including conducting interviews/focus groups, collecting biospecimens, recording anthropometric data) and other research processes ^10, 12, 31– 33, 37, 41, 43, 49, 51, 58^:

The projects have helped me to acquire skills and experience in using mixed methods, in terms of data collection, data entry, data analysis and interpretation. I also acquire excellent skills in report writing and presentation. (p.5: 31).

***New opportunities***

The new skills, competencies and confidence opened up new life opportunities for the CRs including further education and employment:

It’s the first time I’ve had interactions with professors and lecturers and the university campus, and it made us aware we too can come here (p.5: 34)

Future employment ambitions, particularly when working in areas where secure employment was scarce, was a motivating factor to become a CR ^37^. Following their involvement, CRs became motivated to pursue further studies ^49^, to improve their literacy ^23^, communication, or problem-solving skills ^41^ or had goals of gaining employment in specific areas, such as dietetics ^41^, social work ^51^ or accountancy ^49^. In Garnett *et al.*’s study, some CRs went on to become a teacher, a nurse, a mechanic, or to train in agriculture ^49^. Other CRs spoke of wanting to be involved in future research ^23, 49^, or community ^49^ projects. Receiving a certificate verifying their involvement was believed to enhance CRs’ CVs and provide an advantage for future employment ^10, 32, 34^. In one study, the research leads supported the CRs in finding employment after the study had ended ^38^.

***Hearing the stories***

An unintended consequence for some CRs was the negative impact of hearing community-related issues. This sometimes left CRs feeling powerless when confronted with extreme poverty and health-related deprivation ^24, 38^ – as one study described, *‘interacting amidst the economic, cultural and social power inequalities of the community*’ (p. 29: 39). In Mosavel *et al.*’s cervical cancer study undertaken in South Africa, CRs were exposed to a range of serious social problems, including gang affiliations, corporal punishment, drug use, murders, lack of financial and health-related support, and teenage pregnancies ^37^. One CR described the ‘ *big time’* impact of hearing these stories:

Part of my experience while doing this project is that it can be stressful a lot and it’s not just about interviewing people, it’s [what] you hear and see from them. It does affect you because some of the things you hear and see, you may think it’s ok, but when you get home you think, and it starts to haunt you. (p.7: 37)

CRs were reported to experience low moods, shame, shock, helplessness, frustration, fear, disgust, anger or lack of sleep based on the injustices they heard (e.g., 37, 38, 43, 46, 51), and their inability to enforce changes in their communities ^37, 43^. 

Some studies referred to how the wider research terms tried to help address these issues, such as through providing debriefing and supervision sessions ^38, 45, 56^, additional training ^38^ and encouraging the CRs to complete journals ^37, 38, 40^ to reflect upon their experience. Support from a psychologist was reported in several studies (e.g., 10, 37, 38, 50, 56). Although in one study the introduction of a psychologist only occurred after they became aware of the negative impacts on CRs ^37, 38^. 

***Fieldwork hazards***

Fieldwork hazards were reported in several studies. During Mosavel *et al.*’s project exploring mothers and their adolescent daughters’ relationships in the context of cervical cancer in South Africa ^37^, there was a national taxi strike; taxi drivers threatened commuters to prevent them from using public transportation. As CRs were reliant on local transportation to undertake interviews with study participants, the strike not only delayed data collection, but directly exposed CRs to violence in their communities:

Because of the strike I had to postpone [the interviews]. It was scary because they were beating everyone who was traveling, and they burned buses and stoned train carriages. You know a lot of things happened this year. …and they damaged a lot of things and killed a lot of people. In my street a lot of people who were security guards were beaten up and their lives were threatened. (p.8: 37)

Other hazards related to unsafe neighbourhoods ^56^, illness, adverse weather, transportation difficulties, and encountering (or fear of) snakes or ferocious dogs ^32, 34^. CRs could also face refusals, insults and/or demands for money or photographs ^31, 34^. One CR stated:

They [adults in the village] were saying this is nonsense and even swearing at us. (p. 221: 32)

In the Keygnaert *et al.* study - exploring the nature of violence that sub-Saharan migrants experience - two CRs left the study because of receiving security threats ^57^.

***Community benefits***

There was evidence that involving CRs could lead to wider community benefits. For instance, in the study by Brear *et al.* in Swaziland, the knowledge about HIV in their community helped to challenge the CRs stigmatised beliefs, and to promote positive change ^23, 24^. For example, one CR planned to enhance his community-based health work by raising awareness of individuals with HIV having to drink dirty water when taking medication. A further paper from the same study ^13^ highlighted that the CRs, motivated by discussions on the importance of recreation, started under 15s football and netball teams, and raised money for the community via a friendly competition. Other CRs distributed goods (second hand clothes and solar lights) to targeted households (with CRs returning one month later to check the lights worked). The CRs also designed, planned, and hosted a community sports day. Although, important to reflect that while CRs in this study raised other actions they wished to take, these were often large-scale change (e.g., roads, water) that required substantial investment ^13^. 

In Moyo *et al.*’s South African study, the CRs’ new insights about healthy eating, malnutrition and infections was believed to have led to the CRs’ families as well as wider community members indirectly benefiting from this knowledge ^41^. In Burke *et al.*’s study undertaken in Senegal about young people with disabilities, the CRs spoke of wanting to use their newfound knowledge to improve health literacy and outcomes ^36^. Whereas in Price *et al.* the CRs identified areas where the project could be improved, such as where to make condoms more easily available, and acting as an advocate for young people to access health care ^54^. A few projects also described collaborating with statutory and non-statutory organisations to deliver community-based support in response to needs identified through the research. For example, in Schatz *et al.*, support included the distribution of food parcels when necessary ^45^. Similarly, Simon *et al.* compiled a list of available support services which CRs could provide to study participants ^38^.

Brear also referred to how their work had challenged gender norms, by inviting female CRs to speak at community meetings, leading to potential ‘subtle gender-transformative effects’ ^23^. Other ways in which CRs invoked change involved training their peers in research skills, to ‘ *extend the research to different places so we can then address problems’* (p.160: 30). 

### Theme three: Benefits and challenges to the research

In this section, we describe four sub-themes. The first highlights a key benefit of involving CRs - their capacity to access and represent 'unheard voices' in the research. The remaining sub-themes address challenges associated with working with CRs, including issues related to their skills and capabilities, the time and resources required to undertake the research, and the demographic and skill-based barriers they may encounter when engaging with the community.

***Unheard voices***

Several studies highlight how CRs enabled and encouraged community participation (e.g., 12, 36, 43, 47, 51, 56). By virtue of their shared characteristics or backgrounds with study participants, CRs helped to overcome trust barriers and possessed the contextual knowledge that facilitated both access and insight ^38, 45, 49, 51, 55, 56^ - locating entry into the *‘local social and cultural system’* of the community (p.10: 54). 

In Kana *et al.*’s research on street children in the Democratic Republic of Congo, the authors reflected that these children had previously been unwilling to participate in research due to a lack of confidence or fear of potential sanctions (e.g., arrest) ^56^. However, employing CRs who were themselves former street children increased participants' willingness to engage. Some study authors reflected on how the diversity of interviewees was due to recruiting CRs of different genders, ethnicities and socio-economic backgrounds ^45, 54^. CRs also helped to facilitate access between the members of the research team and community gatekeepers (i.e., community elders, ^55^); in the study by Burke *et al.* this involved the research supervisor being introduced to the Presidents of disabilities associations ^36^.

The CRs’ understanding of ‘what works’ in terms of when, where and how to approach participants were reported to have strengthened the quality of data collected ^36, 55^. For example, in Shaw *et al.*’s study exploring renewable energy in Kenya, when the youth groups were not fully engaging, the CRs responded by engaging them in creative activities ^50^. The CRs’ lived experience and ability to more easily create rapport - such as through using ‘local colloquialisms and nuances’ ^45, 54^ - meant the research benefitted from a greater understanding of the participants’ experiences and needs ^35, 40, 43^; to allow the participants’ voices ‘ *to be really heard’* (p. 7: 42). 

Several authors discussed how CRs could uncover issues that participants would not necessarily divulge to academic researchers ^10, 12, 30, 32, 36^. This included highly sensitive information such as being raped or having sex before marriage (in a context where such practice is heavily stigmatised) ^36^. In Burke *et al.*, the CRs referred to using terms such as ‘us’ and ‘we’ to highlight their insider status and to encourage disclosures:

It occurred sometimes that I explained to the informants using “we the disabled” and I felt that this played an essential role in the responses...for example in Kaolack [study site] where I said, “what are the barriers that we, disabled people, encounter when accessing services” ...and the answers flowed wonderfully. (p.9: 36)

CRs were also observed to use strategies to reduce social desirability bias such as asking participants to move location, take short breaks, agreeing terms to discuss culturally sensitive topics, and ensuring participants had been able to express their opinions at the end of the interview ^51^. 

Several studies compared the quality of data collected by CRs with other members of the research team. Ngarachu *et al.* explored students’ attitudes towards ethnicity and politics in two secondary schools in Kenya, comparing the quality of data collected by young CRs with that collected by adult researchers ^58^. Overall, the comparison revealed similar data quality, with differences attributed to the CRs’ questioning style, despite the quantity of data being greater in the adult-led interviews. Robson *et al.*, however, found that young CRs captured more vivid and visceral insights into children’s emotional and physical experiences compared to adult researchers ^33^. The study by Page *et al.* also found that the quality of data collected by CRs improved over time due to supervision and coaching being provided during early data collection ^10^.

Overall, several barriers affected both data collection and the quality of the research, described as follows.

***Skills and capabilities of the CRs***

Challenges related to the skill and capabilities of the CRs were raised in several studies, which on occasion was attributed to the unrealistic expectations being placed on the CRs. For example, in Burke’s study, CRs were tasked with transcribing their interviews – as none had prior experience, this proved challenging, leading to errors and a protracted process ^36^. There were issues of topic guides being followed too rigidly by CRs ^36^, or CRs not probing certain issues ^10, 24, 29, 33, 45, 58^, which the authors considered may be due to discomfort with the subject area, or lack of skills. 

In Page *et al.*’s study exploring rights-based sexuality, the CRs experienced challenges in hearing insights that conflicted with their values and beliefs, leading to lower data quality ^10^. Whereas the CRs in Spuerck *et al.* found keeping young people engaged while encouraging them to discuss sensitive issues during the focus groups was difficult ^12^. A study conducted in The Netherlands and Indonesia found that while CRs were generally less inclined to discuss gender, this reluctance was particularly pronounced in Indonesia - a difference attributed to cultural norms that favour deference to authority and discourage critical questioning ^10^. Similarly, in Brear’s study, CRs were reported to express frustration about not having their answers validated as ‘right’ - a reaction the author attributed to limited critical analysis skills ^23^.

***Engaging with the communities***

Several difficulties related to the CRs’ capacity to engage with community members, categorised into four key areas ‘relationships with community members’, ‘research focus’, ‘demographic-related issues’ and ‘time and resources’.

**Relationships with community members**

Despite the benefits of CRs’ connections and shared experiences with study participants, several studies highlighted challenges related to their engagement with community members. In situations where CRs were known to participants, this familiarity could give rise to mistrust and concerns that they might share confidential information within the community ^27, 45^. 

Additionally, there were assumptions that CRs were already aware of certain information ^27, 51^. As one CR noted: *‘Sometimes they just assumed that I knew some of the facts and their opinions’* (p. 10: 51). Moreover, using known CRs with elevated social status, such as community police officers ^24^ or local ‘gurus’ (head of sex worker networks and important gatekeepers) ^48^ was recognised as having the potential to have influenced what data was collected. While some of the studies introduced strategies to address these challenges – such as CRs not interviewing those overly similar or very well known ^51^ - there were also similar concerns of mistrust and negative impacts on participant recruitment raised in relation to CRs who were *not* known by community members ^12, 32, 50^. In Schatz *et al.*, trust was established by CRs returning to households’ multiple times enabling a CR-participant relationship to form, and to allow for more sensitive conversations to be undertaken ^45^:

We can make promises because everyone knows that we are children, then it’s different from adults, because they can’t hold us responsible. (p.228: 25)

**Research focus**

Challenges of researching sensitive issues such as sexual health in communities where such issues are taboo ^32, 35^ or discussing issues such as ‘sexuality and [pre-marital love] affairs’ when family members were also present ^51^ were highlighted. There were also issues of misinterpretation about the study focus. For example, CRs in Porter *et al.*’s study described how some children were ‘frightened’ by the prospect of having their photo taken, fearing that the CRs were advertising poverty ^32^. One CR from the same study highlighted how the research on mobility issues had been misinterpreted:

There is a lady I attempted to interview. She came to my house to say I had asked about rape questions. My parents explained to her, but she didn’t continue the interview. She was really angry when she came to report this, called me “silly boy”. (p. 8: 32)

There was evidence of husbands ^37^ or parents ^32^ refusing to allow their wives or children to participate in the study:

The parents had a problem because they thought I’d want personal information about income and sleeping around and such and the young children would tell. So, I had to talk to the parents first. (p.221: 32)

CRs were also reported to express disappointment when participants declined to take part ^24, 32, 39^, which for some CRs was thought to stem from a sense that they had ‘ *nothing to offer’* (p.30: 39). 

**Demographic-related issues**

In several studies, the impact of the CRs’ demographic-related characteristics on data collection and quality was discussed. In Burke *et al.*’s study exploring access to sexual and reproductive health services for young people with disabilities, there was evidence of a woman feeling uncomfortable being interviewed by a male rather than a female CR ^36^. While this challenge may be linked to gender norms, one CR suggested it could also relate to a woman’s history of sexual abuse ^32^. However, even in studies not focused on sensitive issues - male CRs sometimes struggled to interview female participants and vice versa – *‘it was easier* [interviewing] *with girls than boys, because I’m afraid, I’m shy of boys’* (p. 220: 32). 

Age related issues were also highlighted. In Robson *et al.*, some young CRs preferred to interview children who were younger as they were perceived to be more compliant ^33^. There was also evidence of CRs feeling threatened by older participants, which raised concerns about the questions being perceived as rude or undermining – ‘[They] *were saying this is nonsense and even swearing at us’* (p.221: 32). Some of the older CRs from the same study were also sceptical about interviewing younger children, questioning their capacity to understand and respond to the questions ^32^. In one study, ethnicity was also highlighted whereby CRs considered that participants were more likely to *‘trust White people’* (p.228: 25).

Some studies also raised concerns that mismatches in characteristics between CRs and the communities they represented - such as differences in privilege, education, language, or ethnicity - could affect CRs’ ability to engage in the research, remain involved, and accurately represent their peers ^30, 31, 50^.

**Time and resources**

A further issue in engaging with study participants concerned time and resource-related issues. Both CRs and study participants could experience conflict when research activities interfered with their domestic chores or other family responsibilities ^24, 30, 33, 34, 51^. On occasion this meant that data collection had to be organised during unsocial hours:

I had to conduct late night interviews because my participants weren’t available during the daytime. . .it was a bit difficult for me. (p. 10: 51).

In a multi-site study ^32– 34^ (Malawi, Ghana, South Africa) involving school-aged CRs, some siblings supported the CRs by taking over their household responsibilities. However, in other cases, this dynamic caused tension or led to attrition from the study. The challenges of coordinating a three-country study also meant that some activities took place during term time. This led to one child being unable to participate, and several children complaining about missing schoolwork:

What I did not like about doing the research is when we are doing our meetings or research while our friends are learning, and I do not want to miss my classes (p. 471: 33).

There were resource-related issues of CRs having to walk for long periods (i.e., up to an hour) ^24^ and limited public transport that impacted on data collection ^37, 56^. In two studies, the authors highlighted how unanticipated resources had been needed to support the CRs (e.g. in additional training and support) ^37, 50^. There were also wider practical challenges of CRs and participants experiencing discomfort and difficulties with video calls ^51^ and equipment (cameras) not working ^32^. In Mosavel *et al.*’s cervical cancer prevention study, the actual workload and administrative aspects of conducting research caused some of the CRs *‘concern and stress’* (p.7: 37).

### Theme four: ethics of engagement

In this theme, we describe different ethics-related issues in terms of how the research was undertaken (‘ethical considerations’), ‘power differentials’ amongst CRs, participants, research teams and wider community members, and ‘paying the CRs’.

***Ethical considerations***

One study involved working with the CRs to develop a code of conduct for engaging with study participants ^31, 33^, covering issues such as respect, preventing harm, the voluntary nature of participation, informed consent and flexibility in data collection. A further code of conduct was described in the paper by Rink, but this related to how the CRs would work together ^44^. The shared rules - collectively agreed upon - covered issues such as session timing, active listening, mutual respect, ensuring all voices were heard, equality, and mobile phone use. 

In a few studies, the research team stipulated boundaries in data collection, to protect CR wellbeing and to enhance data quality. These stipulations included CRs not recruiting from their immediate communities ^45, 51^, or family members, should they feel uncomfortable to do so ^45^, and in the Burke *et al.* ^36^ and Simon *et al.* ^38^ studies, to not recruit anyone they knew. 

In some studies, CRs conducted interviews in pairs (e.g., 13, 34, 37) largely designed to enhance the CRs’ safety. In Mosavel *et al.*, the CRs could take a ‘ *protector*’ with them (with a stipend provided), and CRs were given clear instructions to terminate the interview should any safety issues emerge ^37^. The CRs in Schatz *et al.* could also request the support of another CR as needed ^45^. In several studies, the CRs were accompanied by a member of the research team who would support or oversee research related procedures such as data collection and consent (e.g., 13, 55): in Kana *et al.*’s study, the psychologist who was present during interviews only intervened if the CR missed an important question ^56^.

Other ethics-related issues concerned confidentiality. In Schatz *et al.*’s study, the CRs were tasked with having public rather than private conversations, to avoid discussing personal or sensitive issues ^45^. In Elmusharaf *et al.* the CRs did not record personal information about the participants: a strategy designed to encourage participation ^52^. Two studies ^48, 52^ adopted a more unique perspective of using a third person interviewing approach, whereby participants were asked to comment on the behaviours of others in their networks. This approach was believed to allow participants to avoid accounting for their own behavior, to protect against individuals feeling vulnerable, and allowing different and conflicting viewpoints to emerge. In one study, the CRs were asked to sign data confidentiality clauses to confirm they would not disclose any data ^35, 36^.

A further ethics-related challenge related to the literacy of study participants ^38, 51^. In Simon *et al.*’s study exploring cervical cancer, participants were reported to not understand the purpose of the study, requiring multiple iterations of providing study information, and participants answering questions not always related to the research ^38^. This challenge was addressed by co-developing a pre-consent quiz to use with participants. The questions included ‘can you tell me why you are interested in this research?; can you tell me what this research is about?; can you tell me what you are being asked to do for this research?; and provided opportunities for the participants to ask further questions. The introduction of this tool was found to make a positive impact on recruitment.

The issue of providing incentives to participants was raised in a few studies. While gifts to participants were condoned in some studies ^53^, other studies referred to CRs facing personal difficulties ^34^ or hostility when this was not provided ^50^. One CR reported:

If I asked them for permission they said, can I give them money as they haven’t eaten, and some wouldn’t let their children talk to me (p.222: 32).

In Brear’s study, CRs suggested that items such as soap or food should be offered as a gesture of appreciation ^24^. However, they also expressed concern about the potential for coercion. As a compromise, it was agreed that these items would be provided only after data collection had been completed to thank them for their involvement, rather than incentivize their involvement ^24^.

A final issue concerned protecting CRs from inaccurate information. In Elmusharaf *et al.*’s project CRs were given evidence-based insights about maternal health issues, to counter act any personal risks of receiving misleading or incorrect information from community members ^52^.

***Power differentials***

Power differentials regarding consent, demographics and team working were discussed in several studies. In some of the studies, this related to recruiting CRs who were more educated ^58^, from positions of authority ^24^, or those who have little autonomy (i.e., children) ^30^. Power differentials also related to gender related norms. In a few studies ^24, 45^, a two-stage consent process was established in line with local values, whereby men were contacted first, and asked for permission to speak with their wives:

[The respondent’s] husband came and said, “Don’t talk to my wife. Tell me what you want.‟ I explained why I wanted to talk to his wife. Then he allowed me. When I finished the interview, I thanked the husband, and he said that from now I may come any time to talk to his wife. (p.380: 45)

Power-related issues were also identified within the research teams, often associated with the CRs’ gender, age ^40, 43, 50, 53^, or the CRs having stigmatised sexual identities ^48^. Some of the ways teams worked to address these issues included involving the CRs in data collection tools and processes (e.g., 33, 40, 43). Other strategies involved using terms such as ‘debrief buddies’ rather than ‘supervisory meetings’, having an open-door policy, privileging local languages ^51^ and encouraging the CRs to share their knowledge within the research team ^44^. While in Brear’s study ^23^ it was felt that the CRs’ relationship within the research team shifted from a position of adult–child to one of a more equal footing – in Robson *et al.*’s study the authors felt that ‘ *despite our efforts, we remain at times uncomfortable with the power relations enmeshed in our research’* (p.470: 33). 

Wider cultural norms led some gatekeepers to dismiss or undervalue the role of young CRs, particularly when the research addressed sensitive topics such as sexuality. These subjects were often considered inappropriate for young people, who were perceived as too sensitive or for whom such discussions were deemed culturally off-limits ^10^. Some authors also spoke of the challenges of advocating for children’s rights and involving young CRs in countries- such as Ghana - where children's roles and rights are less valued ^30^. 

Some young CRs faced hostility ^10^ or ambivalence ^32^ from stakeholders, which on occasion required intervention by senior adult staff ^10^. These authors stressed the need for careful groundwork in engaging with key influential stakeholders to build stakeholder-CR relationships, and for the work and findings to be acknowledged ^10, 30, 32^. When young CRs in Porter *et al.*’s study presented their findings to a wider stakeholder group, this was believed to have helped age-related biases ^32^. However, in Page *et al.*’s study, stakeholders in Indonesia requested that the Principal Investigator - rather than the young CRs - present the findings ^10^. This reflected prevailing cultural values and perceptions regarding academic authority and formal qualifications.

***Paying the CRs***

While providing payments to CRs is standard practice in the UK and other Western contexts, in several of the included studies, this issue was not discussed (e.g., 35, 36, 40– 42, 45, 48, 50, 51, 54, 56, 58). However, even when this information was reported, there were wide variations in both the amounts paid and the processes used to determine them. 

In Brear’s study the CRs received a stipend (of unknown amount) which was believed to validate their perception of themselves ‘ *as important people*’ (p.10: 23). On some occasions, the payment was agreed with local statutory or community groups (e.g., 38, 53, 55) or even agreed by the CRs ^57^. In some studies, there was consideration of payment that reflected local salaries ^10, 25, 38, 55^. Others specified the amount paid to CRs, but with no justification for how the cost had been agreed (e.g., 57), or referred to paying an amount, without specifying what the amount was (e.g., 44, 49). Authors from one of the studies ^44^ reflected that, since all other team members were being paid, it was important to ensure CRs were also compensated to promote equity within the team. 

In Elmusharaf *et al.*’s study, the young CRs were reimbursed the costs of attending the research training and to undertake data collection and were provided with food and accommodation when attending the study workshops, rather than a payment per se ^52^. Young CRs could also receive benefits besides monetary payments such as skill-based training, meals, treats (sweets, films) and a wristwatch ^32, 34^. In the multi-site study on mobility and transportation issues, there was a protracted discussion of various considerations, including a review of employment laws, before deciding to pay the child CRs a wage considered *‘fair though not generous*’ (p. 472: 33), but with *‘some recognition of individual effort’* (p. 224: 32). However, during data collection, unanticipated financial issues arose - such as a CR hiring a bicycle to reach a study site, needing soap after a long and muddy walk home from fieldwork, and having to self-fund travel to attend training workshops. While the authors expected the CRs to cover these additional costs from their ‘wages’, they later regretted this decision, as it resulted in the CRs being economically disadvantaged by their participation ^33^.

The payments received by CRs were highly valued ^34^, and for some, represented a more regular income than they had previously earned ^41^. These earnings supported both personal and familial advancements. Personally, CRs used the funds to purchase school textbooks ^34^, invest in further education ^31, 41, 49^, and pay for driving lessons ^41^. Familial benefits included purchasing essential items such as fertiliser ^34^, refrigerators, stoves, satellite televisions, microwave ovens or towards larger projects such as building or extending houses ^41^.

## Discussion

To our knowledge, this is the first synthesis of existing research to explore how community researchers (CRs) are recruited and supported, and the experiences, challenges and barriers of using CRs in LMIC settings. Overall, many of our findings resonate with those obtained in high-income countries (HICs). From a positive perspective, CRs are often trusted insiders, who facilitate access to marginalised groups and help reduce power differentials between participants and research teams; their involvement can lead to the collection of more authentic and culturally relevant data; and participation in research can enhance CRs’ confidence, build social capital, open up future employment opportunities, and foster broader community change ^2, 59– 63^. From a negative perspective, CRs may lack the necessary research rigour and methodological skills, and their personal biases can sometimes conflict with broader community values; participants may be reluctant to share sensitive information due to concerns about confidentiality; CRs may experience emotional distress from exposure to traumatic narratives, and their limited involvement beyond data collection stages can result in tokenistic participation ^2, 60, 62– 67^. However, despite these similarities and differences between the experiences of CRs in HICs and LMICs, there are key ethical issues of using CRs in settings affected by poverty, poor literacy, unemployment, gender norms and age-related prejudices which CRs working in HICs would not necessarily be exposed to. In the following section, we consider these wider challenges in the context of how CRs are recruited, trained and supported when using this participatory approach.

Across the studies, the most common form of recruitment method was to advertise within existing community, statutory or non-government organisations (e.g., 12, 30, 38, 46– 48, 52, 53, 55, 56, 58). This approach offers clear advantages, as these organisations are often trusted within the community and may already have established relationships with, and knowledge of, the CRs ^63, 68^. However, across the included studies, CRs appeared to be more commonly recruited based on their sociodemographic characteristics rather than personal attributes such as communication skills or critical thinking abilities. A lack of consideration for the capabilities of CRs could lead research teams to compromise on data collection methods ^50^, while also preventing CRs from participating in data analysis as originally intended ^35, 36^. In some of the studies there were criticisms regarding the skills and capacities of CRs negatively impacting the quality and integrity of data collection ^10, 12, 23, 27, 36, 45, 58^. These critiques highlight the importance of implementing a more meaningful and sensitive recruitment process- one that includes mechanisms for assessing the capabilities, readiness, and support needs of prospective CRs. 

However, the challenges associated with the skills and capabilities of the CRs also draws into question the training and support provided. Overall, it was difficult to compare the training programmes across the studies. While a few studies offered rich descriptions ^13, 52^ or were guided by existing frameworks (e.g. 10, 35, 36), the majority provided only brief overviews of in-house training programmes that were typically focused on research-related processes and procedures, offering limited insight into the depth or scope of CR capacity-building. 

Role-play was a common approach within the training – an approach that enables participants to ‘learn by doing’ by developing verbal and non-verbal communication, to promote empathy, and to prepare for challenges ^69, 70^. Only one study adopted a more flexible training approach, in which role play continued until the CRs were deemed sufficiently skilled ^51^. Furthermore, while most projects offered supervision, mentoring, and/or debriefing sessions, it was often unclear whether these were used to assess the quality of the data collected or to support the CRs. The fact that CRs continued to face challenges suggests that more sustained opportunities for skill development, along with feedback and mentoring based on actual data, would be beneficial.

Furthermore, CR involvement was often limited to the development of data collection tools rather than full engagement across all stages of the research process. This pattern reflects broader critiques of tokenism, which risks reinforcing existing power imbalances and undermining the credibility of the research ^11, 71^. To address these concerns, greater efforts are needed to ensure the full integration of CRs, facilitated by appropriate training and ongoing support. Notably, none of the studies reflected on CRs’ experiences of the training itself - highlighting a key evidence gap. Future studies should explore the potential of co-developing and evaluating CR training programmes in collaboration with the CRs themselves.

Across several studies there were issues related to how gender, sexuality and age-related norms could impact on the research – issues unlikely to be as prevalent in Western contexts. There were issues of CRs ^32^ and study participants ^24^ being potentially compromised to take part and CRs feeling uncomfortable collecting data from those of a different gender ^32, 36^ or age ^32, 33^. Difficulties were also noted in researching sensitive subject areas such as sexual health, particularly in countries where such practices were taboo ^10, 35, 51^. Studies could also face patriarchal-related challenges of husbands not allowing their wives to participate ^37^, and the research findings being potentially devalued as it involved children and young people as data collectors ^10, 30, 32^.

The CRs sociodemographic characteristics were also noted to cause tension and conflict within the research teams ^40, 43, 48, 50, 53^, with these power differentials not always being fully addressed ^33^. Therefore, while matching CRs to the study population offers clear benefits in terms of access and the depth of data collected, the problems highlighted emphasise a need for more deliberate consideration before, during, and after the study. These considerations should include who CRs should approach when they experience challenges, and careful preparation of the research team and wider stakeholders. Such reflection is essential to ensure that the priorities of CRs, the research objectives, and the needs of the wider community are meaningfully addressed.

The benefits for CRs in LMICs may be even more impactful due to the relative scarcity of resources and opportunities, making such roles especially valuable for personal and community development. Across several of the studies, CRs spoke of how their involvement had led to an improved self-identity, positive impacts on social relationships, altered perceptions about their own lives, reduced prejudices, and better preparation for future adversity ^36, 40– 44, 46, 49– 51, 55, 57^. Importantly, working as a CR offered new skills, competencies and confidence to pursue new educational or work-related opportunities ^10, 23, 32, 34, 37, 41, 49^ thereby granting access to resources and opportunities that might otherwise have been unavailable to them. 

However, from a critical perspective, participation in research also carried the potential for harm to the CRs. CRs being negatively affected by hearing the stories of community members is reported in the wider literature ^9, 11, 72^. However, when focused on studies in LMICs, the potential for harm appears to be more nuanced and pervasive. First, the stories that CRs were exposed to, on occasion unrelated to the research topic, could be extreme, including gang violence and rape ^37^ – hearing about others’ misfortunes was associated with a whole array of negative emotional responses (e.g., 37, 38, 43, 46, 51). 

Other harms related to exposing CRs to fieldwork hazards, such as community violence, unsafe neighbours, adverse weather, transportation difficulties (including travelling for long periods of time for data collection purposes) as well as threats of harm from community members ^31, 32, 34, 56, 57^. While some authors reported attempts to address these issues - through interviews being undertaken in pairs, CRs accompanied by members of the research team, training and role play, supervision or debriefing, or community researcher-led activities such as journal writing (e.g., 13, 34, 37, 38, 40, 45, 56) - the therapeutic value of these strategies was unclear. In one study, remedial actions were only introduced after the emotional impact on CRs became evident ^37, 38^, suggesting a lack of prior insight into the communities they intended to research. These issues raise important ethical concerns about the harms and benefits to the CRs. Clear consideration of all potential risks and how they will be minimised should be maintained throughout. Opportunities for debriefing - particularly with individuals trained in this approach - should also be included to ensure that participation in research does not exacerbate health inequalities or lead to poorer outcomes for those who are already disadvantaged ^73^.

A final issue relates to the financial or social costs of involving CRs, where inconsistent or insufficient consideration can exclude economically disadvantaged individuals and inadvertently devalue their expertise ^63, 74^. In the UK, like other Western contexts, paying peer researchers is standard practice, and is meant to flatten power dynamics and create equal partnerships ( www.involve.co.uk). However, our review highlights a concerning gap in the literature, with many studies providing no detail or reference to whether CRs in LMICs received any form of payment. Even when payment was acknowledged, details concerning the amount and the rationale for it were often omitted (e.g., 13, 23, 24, 39, 49). There were also instances where CRs incurred out-of-pocket costs that had not been anticipated or factored into the project budget ^33^. Children and young people serving as CRs often have school, household and potential employment responsibilities that contribute to supporting their families. Their involvement in research could therefore lead to conflicts and increase the risk of attrition ^32– 34^. As payments received by CRs were highly valued and often used to support both personal and familial needs ^34, 41, 49^, research teams should ensure that compensation is fair, equitable, and reflective of the value, importance, and equality of their role.

## Conclusion

This review represents the first synthesis of how community researchers are recruited, trained, and supported in low- and middle-income country (LMIC) settings, highlighting both the benefits and pitfalls of this participatory approach. While CRs can improve access, cultural relevance, and authenticity in research, their involvement also raises significant concerns around ethical practices, training quality, emotional wellbeing, and equitable compensation. These issues are compounded in LMIC contexts by structural inequalities, limited resources, and sociocultural challenges. To maximise the benefits and minimise potential harms, research teams should adopt more thoughtful and inclusive approaches - ensuring meaningful recruitment, robust training, sustained support, and fair remuneration. Ultimately, for CRs to be truly empowered rather than tokenised, their contributions must be valued as integral to the research process, not peripheral.

## Data Availability

This is a secondary review of existing published research. The data for this article consists of bibliographic references, which are included in the References section. An extended data set has been created: UCLanData: Engaging and supporting Community Researchers in Low and Middle-Income Countries: An Integrative Review. https://doi.org/10.17030/uclan.data.00000623 (Thomson *et al.*, 2025) This project contains the following extended data: - Supplementary File 1_Search strategies.docx (Search strategies) - Supplementary File 2_Community Researchers PRISMA_2020_checklist.docx (PRISMA Checklist) - Supplementary File 3_Table 1 Study community researchers and study participant characteristics.docx (Table 1: Study community researchers and study participant characteristics) - Supplementary File 4_Table 2 Recruitment, involvement and training of community researchers.docx (Table 2: Recruitment, involvement and training of community researchers) Data are available under the terms of the Creative Commons Zero “No rights reserved” data waiver (CC0 1.0 Public domain dedication).
