# Supplementary material for: Engaging and supporting Community Researchers in Low and Middle-Income Countries:  An Integrative Review
Source: Wellcome Open Res. 2026 Jun 3;10:531. Originally published 2025 Oct 1. [Version 4] doi: 10.12688/wellcomeopenres.24827.4 (PMC12648019; doi:10.12688/wellcomeopenres.24827.4)
Supplement: Peer review history

# Reviewer report: Reviewer response for version 4

**DOI:** 10.21956/wellcomeopenres.28894.r157237

**Published:** 2026-06-05

**Authors:** Liz Pellicano

**Affiliations:** 1 University College London, London, UK

I thank the authors for addressing my additional comments. I have no further comments.

Is the review written in accessible language?

Yes

Are all factual statements correct and adequately supported by citations?

Yes

Are the conclusions drawn appropriate in the context of the current research literature?

Yes

Is the topic of the review discussed comprehensively in the context of the current literature?

Yes

Reviewer Expertise:

Autism research; participatory research of marginalised communities

I confirm that I have read this submission and believe that I have an appropriate level of expertise to confirm that it is of an acceptable scientific standard.

# Reviewer report: Reviewer response for version 3

**DOI:** 10.21956/wellcomeopenres.28599.r149064

**Published:** 2026-03-10

**Authors:** Liz Pellicano

**Affiliations:** 1 University College London, London, UK

I thank the authors for addressing my comments and concerns. I am generally satisfied with the revisions but there are a few outstanding issues I wish to raise here.

 First, it was good to see the inclusion of Cohen’s Kappa for the quality appraisal. But I couldn’t see its inclusion for the first stage (title + abstract) and second stage (full-text review) screening. Including the degree of inter-reviewer agreement would strengthen the quality of the review.

 Second, I thank the authors for clarifying their analytic process – which is based on Thomas and Harden’s thematic synthesis approach, rather than reflexive thematic analysis. That said, even in the case of a thematic synthesis, it is important for authors to outline their epistemological stance – to ensure transparency, rigour and for the reader to understand precisely how knowledge is being constructed from the primary studies. Could the authors please include more details on their epistemological stance?

 Finally, it is also great to see a positionality statement. I note, however, that none of the authors are from LMICs and only one has worked in community-based research in LMICs. Given that the authors highlight the lack of community involvement in the studies eligible for review, it would be prudent also to note the lack of community involvement in the systematic review itself. I suggest the authors include this as a potential limitation of their work.

Is the review written in accessible language?

Yes

Are all factual statements correct and adequately supported by citations?

Yes

Are the conclusions drawn appropriate in the context of the current research literature?

Yes

Is the topic of the review discussed comprehensively in the context of the current literature?

Yes

Reviewer Expertise:

Autism research; participatory research of marginalised communities

I confirm that I have read this submission and believe that I have an appropriate level of expertise to confirm that it is of an acceptable scientific standard, however I have significant reservations, as outlined above.

# Response

**Published:** 2026-03-10

**Authors:** Gill Thomson

**Affiliations:** School of Nursing & Midwifery, University of Central Lancashire School of Community Health and Midwifery, Preston, England, UK

Many thanks for re-reviewing our paper. In response to your comments, we will revise the manuscript to include further detail regarding our epistemological stance. While community involvement was not a central focus of our critique, we recognise the importance of reflexivity in global health research. In response to your suggestion, we will add additional text to the reflexivity section acknowledging the limitations related to the composition of the research team. Concerning Cohen's kappa, as two reviewers screened all hits at title + abstract and full-text, we followed PRISMA guidelines (#8 – Page et al., 2021) by reporting how many reviewers screened the studies, whether screening was independent, and how disagreements were resolved.  We hope this resolves this issue.

# Response

**Published:** 2026-05-06

**Authors:** Gill Thomson

**Affiliations:** School of Nursing & Midwifery, University of Central Lancashire School of Community Health and Midwifery, Preston, England, UK

Many thanks for the further comments - all these revisions have now been addressed.

# Reviewer report: Reviewer response for version 2

**DOI:** 10.21956/wellcomeopenres.28134.r144299

**Published:** 2026-02-11

**Authors:** Sonja Klingberg

**Affiliations:** 1 Karolinska Institute Department of Learning Informatics Management and Ethics (Ringgold ID: 411412), Stockholm, Stockholm County, Sweden

This is an interesting systematic search and integrative review examining literature on how community researchers in LMIC settings are engaged and supported. It includes a comprehensive search, screening and quality appraisal process, and reports using reflexive thematic analysis for synthesising findings from included studies that were all qualitative.

 The review process and reporting have been addressed by other reviewers and updated by authors as far as I can tell so my comments focus on the choice to focus on LMICs and the qualitative analysis/synthesis.

 It is not made clear to the reader why the scope of the review is defined by the classification of countries by income. There has been critical discussion on the use and usefulness of such categorisations in e.g., BMJ Global Health (see Khan et al. 2022 "How we classify countries and people—and why it matters" and Lencucha & Neupane 2022 "The use, misuse and overuse of the ‘low-income and middle-income countries’ category") and while I do not propose changing the scope at this stage, I do think it is important to acknowledge its limitations and assumptions. Since the discussion section does not currently engage much with the limitations and strengths of this review itself, it would be something that can be included there, in addition to ideally providing a clear rationale in the introduction.

 Another recent peer review of this article by Liz Pellicano captured many of the observations I was also about to make about using and reporting the use of reflexive thematic analysis so I will not repeat those. Nevertheless, I echo the assessment that the themes seem rather descriptive or under-developed. I wanted to therefore suggest the possibility of revisiting the choice of analysis method. It is not necessary to use reflexive thematic analysis if it is not the most suitable choice for your purposes. I recommend reading Braun and Clarke's writing (including their website www.thematicanalysis.net) on different types of thematic analysis, and perhaps considering other methods such as more deductive or descriptive variations of thematic analysis or something different like framework analysis methods. However, if you maintain that reflexive thematic analysis is the best choice for this purpose then you do need to provide more of a justification, details and nuance as described in the peer review report by Liz Pellicano.

Is the review written in accessible language?

Yes

Are all factual statements correct and adequately supported by citations?

Yes

Are the conclusions drawn appropriate in the context of the current research literature?

Yes

Is the topic of the review discussed comprehensively in the context of the current literature?

Partly

Reviewer Expertise:

Community engagement, public health interventions, qualitative research, process evaluation, realist evaluation.

I confirm that I have read this submission and believe that I have an appropriate level of expertise to confirm that it is of an acceptable scientific standard, however I have significant reservations, as outlined above.

# Response

**Published:** 2026-03-10

**Authors:** Gill Thomson

**Affiliations:** School of Nursing & Midwifery, University of Central Lancashire School of Community Health and Midwifery, Preston, England, UK

Many thanks for reviewing our article. We have made substantial revisions to the themes and have aligned our work with Thomas & Harden’s thematic synthesis approach following Liz Pellico’s review. The new version (Version 4) also addresses your points regarding the rationale for focusing on LMIC settings and includes limitations associated with this focus.

# Reviewer report: Reviewer response for version 2

**DOI:** 10.21956/wellcomeopenres.28134.r144300

**Published:** 2026-01-30

**Authors:** Liz Pellicano

**Affiliations:** 1 University College London, London, UK

**Thomson et al. Engaging and supporting community researchers in low and middle-income countries: An integrative review**

 I am reviewing this manuscript for the first time. I am aware that the authors have already done one round of revisions, which have addressed some of my concerns, so I will keep this review relatively brief.

 My main concerns are (1) the absence of some methodological details, (2) the analytic process and (3) the resulting thematic structure.

 First, while the methods are generally well described, I was surprised that no details were provided for each stage of the screening process (title and abstract screening; full-text screening) and the quality assessment. Can the authors please provide % agreement and Cohen’s Kappa for each of these stages?

 Second, the authors state that they used reflexive thematic analysis but only provided details of the ‘mechanics’ of the analysis. Reflexive thematic analysis is a very broad – and theoretically flexible – technique, which usually requires a number of decisions to be made. The authors don’t really describe any of these. For example, what framework did they use (e.g. constructionist, realist)? Did they adopt an inductive, deductive, or hybrid approach? Did they code at the semantic or latent level? Analysis is also a situated and interactive process, reflecting the data, the positionality of the researcher, and the context of the research itself. It would therefore be helpful to acknowledge researcher positionality and how the authors engaged in reflexivity throughout the analytic process.

 Also, given that all of the studies were qualitative, the readers need to know more about exactly what the authors coded. I assume they coded both first-order (derived directly from people’s quotations) and second-order results (authors’ interpretations)? This needs to be clearer.

 Third, the analysis itself felt a little superficial in nature. In reflexive thematic analysis, the researcher’s role is to provide a rich, contextualized narrative as to why the themes matter. As a reader, I therefore expected to see a rich, nuanced, and engaging analysis that addresses the researchers’ research questions. Instead, the results read as a series of sections describing each theme/subtheme that are relatively unconnected to one another. Furthermore, all of the theme titles sound more like categories or codes than themes. For example, the subtheme label, ‘recruitment strategies’, tells us very little about what actually comes under this subtheme). I would encourage the authors to re-think their analysis, writing it up precisely as Braun and Clarke propose – intriguing and engaging the reader while at the same time convincing them of the validity and robustness of the arguments and the themes the researcher has identified.

 Finally, one issue that I can’t see has been discussed related to reporting of community researchers’ involvement. The authors note in several places in the methods/results that such reporting was lacking. Perhaps the authors could suggest the use of the GRIPP2 reporting checklist for community involvement, which might be one way for authors to address this limitation in future? https://www.bmj.com/content/358/bmj.j3453

Is the review written in accessible language?

Yes

Are all factual statements correct and adequately supported by citations?

Yes

Are the conclusions drawn appropriate in the context of the current research literature?

Yes

Is the topic of the review discussed comprehensively in the context of the current literature?

Yes

Reviewer Expertise:

Autism research; participatory research of marginalised communities

I confirm that I have read this submission and believe that I have an appropriate level of expertise to confirm that it is of an acceptable scientific standard, however I have significant reservations, as outlined above.

# Reviewer report: Reviewer response for version 2

**DOI:** 10.21956/wellcomeopenres.28134.r144120

**Published:** 2026-01-08

**Authors:** Anthony Philip Coetzer-Liversage

**Affiliations:** 1 ServeMinnesota, Minneapolis, Maryland, USA

No further comments.

Is the review written in accessible language?

Yes

Are all factual statements correct and adequately supported by citations?

Yes

Are the conclusions drawn appropriate in the context of the current research literature?

Yes

Is the topic of the review discussed comprehensively in the context of the current literature?

Yes

Reviewer Expertise:

Implementation Science, Public Health, Mental Health, and Behavioral Health

I confirm that I have read this submission and believe that I have an appropriate level of expertise to confirm that it is of an acceptable scientific standard.

# Reviewer report: Reviewer response for version 1

**DOI:** 10.21956/wellcomeopenres.27350.r138498

**Published:** 2025-11-25

**Authors:** Anthony Philip Coetzer-Liversage

**Affiliations:** 1 ServeMinnesota, Minneapolis, Maryland, USA

**Reviewer Report**

**1. Summary of the Manuscript**

 This integrative systematic review synthesizes evidence on how community researchers (CRs) are recruited, trained, and supported in research projects across low- and middle-income countries (LMICs). The review also explores facilitators, challenges, impacts, and CRs’ experiences. Searches were conducted across seven databases without date or language restrictions, supplemented by citation tracking. Thirty-nine papers reporting 27 studies were included. Data were analyzed using a reflexive thematic approach, and quality was assessed using the Mixed-Methods Appraisal Tool (MMAT). Findings are organized under four themes: (1) recruitment, engagement, and support; (2) benefits and challenges for CRs and communities; (3) benefits and challenges for research; and (4) ethics of engagement. The review highlights benefits such as improved access to marginalized groups and culturally relevant data, alongside challenges including ethical concerns, emotional distress, and inconsistent remuneration. Recommendations emphasize the need for robust recruitment, training, and support strategies to ensure meaningful and ethical involvement of CRs.

**2. General Assessment**

**Originality & Significance:**

 The manuscript addresses an important gap in participatory research literature by focusing on LMIC contexts, where structural inequalities and resource constraints pose unique challenges. This synthesis is timely and relevant for global health and social research.

**Strengths**

- Comprehensive search strategy across multiple databases and inclusion of diverse study designs.
- Clear articulation of objectives and systematic approach following PRISMA guidelines.
- Rich thematic synthesis offering nuanced insights into ethical, practical, and methodological issues.

**Weaknesses**

- Limited critical appraisal of how methodological quality influenced findings.
- Discussion could better integrate implications for practice and policy with concrete, actionable recommendations.
- Some redundancy in presenting findings and discussion; tighter synthesis would improve readability.

**Alignment with Journal Scope**

 The manuscript aligns well with Wellcome Open Research’s focus on global health, ethics, and participatory approaches.

**3. Specific Comments**

**Title:** Accurate and descriptive.

**Abstract**

- Provides a clear overview of aims, methods, and findings.
- Could be more concise; and emphasize novelty and implications.
- Include MMAT as part of quality appraisal in the abstract for transparency.

**Introduction**

- Strong rationale for the review and clear identification of gaps.
- Literature coverage is comprehensive but wordy; condense repetitive explanations of CR roles and benefits.
- Integrate ethical considerations earlier to frame the significance of the review.
- Research questions are clear but could be highlighted in bullet points for readability.

**Methods**

- Adherence to PRISMA and PROSPERO registration is commendable.
- Provide more detail on:
  - How MMAT scores influenced interpretation of findings.
  - Reflexive thematic analysis process (e.g., coder triangulation, software used).
- Clarify exclusion criteria for grey literature and justify its omission.
- Consider adding a summary table of included studies in the main text (currently in supplementary files).

**Results**

- Well-structured thematic presentation with illustrative examples.
- Figures and tables (e.g., PRISMA diagram) are appropriate but captions could be more descriptive.
- Explicitly link findings to review objectives for clarity.

**Discussion & Conclusion**

- Discussion effectively contrasts LMIC findings with HIC literature and addresses ethical complexities.
- Needs stronger integration of findings with practical recommendations:
  - Specify strategies for recruitment (e.g., attribute-based selection).
  - Outline minimum standards for training and supervision.
  - Suggest frameworks for equitable remuneration.
- Conclusion is clear but could be more concise and avoid repeating earlier points.

**References**

- Extensive and current, covering both foundational and recent literature.

**4. Language & Style**

- Academic tone is appropriate, but the manuscript tends to be wordy in several sections (Introduction, Discussion).
- Improve sentence conciseness and paragraph transitions.
- Avoid colloquial phrases (e.g., “big time impact”) in quoted material unless essential for authenticity.
- Ensure consistent tense usage (past for methods/results; present for discussion).

**5. Final Recommendation**

**Recommendation:** **Minor Revision**

**Justification:**

 The manuscript is well-conceived, methodologically sound, and addresses a critical gap in participatory research in LMICs. However, revisions are needed to improve clarity, conciseness, and practical applicability of recommendations. Specifically:

1. Streamline abstract, introduction, and discussion to reduce redundancy.
2. Enhance methodological transparency regarding MMAT scoring and thematic analysis.
3. Strengthen actionable recommendations for recruitment, training, and remuneration.
4. Edit for language clarity and reference formatting.

Upon addressing these points, the manuscript will make a valuable contribution to the literature on ethical and effective engagement of community researchers in LMIC contexts.

Is the review written in accessible language?

Yes

Are all factual statements correct and adequately supported by citations?

Yes

Are the conclusions drawn appropriate in the context of the current research literature?

Yes

Is the topic of the review discussed comprehensively in the context of the current literature?

Yes

Reviewer Expertise:

Implementation Science, Public Health, Mental Health, and Behavioral Health

I confirm that I have read this submission and believe that I have an appropriate level of expertise to confirm that it is of an acceptable scientific standard.

# Reviewer report: Reviewer response for version 1

**DOI:** 10.21956/wellcomeopenres.27350.r135072

**Published:** 2025-10-25

**Authors:** Melissa Petrakis

**Affiliations:** 1 Monash University, Melbourne, Melbourne, Australia

This is a well-written manuscript regarding engaging and supporting Community Researchers (CRs). While the authors are based in the United Kingdom, the study focus is on low and middle income country (LMIC) experiences.

 A comprehensive 7 databases were searched. Overall 39 papers were included, reporting on 27 studies. Clear PRISMA diagram.

 Findings were that the benefits of including CRs were access to marginalised groups, reducing power differentials between participants and researchers, and eliciting more nuanced culturally relevant data. For CRs’ benefits were enhanced confidence, expanded future employment opportunities, and possible positive community changes. Concerns raised were around ethical practices, including possible negative emotional impacts on CRs, and equitable remuneration, particularly in LMIC contexts where there are structural inequalities and limited resources, including for training and support, and sociocultural challenges in the environment. The impact of hearing painful stories of one's community while feeling powerless to change the reality can indeed be detrimental - this is well discussed.

 There is a good coverage in Discussion regarding issues of tokenism rather than genuine coproduction to date in the use of CRs. There is potential to extend research in this area and to use this current study as a benchmark to compare future literature to.

 Very extensive referencing (74 references listed), including throughout the report whereby the reader can follow which studies relate to which specific findings.

Is the review written in accessible language?

Yes

Are all factual statements correct and adequately supported by citations?

Yes

Are the conclusions drawn appropriate in the context of the current research literature?

Yes

Is the topic of the review discussed comprehensively in the context of the current literature?

Yes

Reviewer Expertise:

mental health, codesign, coproduction

I confirm that I have read this submission and believe that I have an appropriate level of expertise to confirm that it is of an acceptable scientific standard.

# Response

**Published:** 2025-10-25

**Authors:** Gill Thomson

**Affiliations:** School of Nursing & Midwifery, University of Central Lancashire School of Community Health and Midwifery, Preston, England, UK

Many thanks for the positive feedback
